# Neuroglobin, a pro-survival player in estrogen receptor *α*-positive cancer cells

**DOI:** 10.1038/cddis.2014.418

**Published:** 2014-10-09

**Authors:** M Fiocchetti, M T Nuzzo, P Totta, F Acconcia, P Ascenzi, M Marino

**Affiliations:** 1 Department of Science, University Roma Tre, Viale Guglielmo Marconi 446, I-00146 Roma, Italy; 2 Interdepartmental Laboratory of Electron Microscopy, University Roma Tre, Via della Vasca Navale 79, I-00146 Roma, Italy

## Abstract

Recently, we reported that human neuroglobin (NGB) is a new player in the signal transduction pathways that lead to 17*β*-estradiol (E2)-induced neuron survival. Indeed, E2 induces in neuron mitochondria the enhancement of NGB level, which in turn impairs the activation of a pro-apoptotic cascade. Nowadays, the existence of a similar pathway activated by E2 in non-neuronal cells is completely unknown. Here, the role of E2-induced NGB upregulation in tumor cells is reported. E2 induced the upregulation of NGB in a dose- and time-dependent manner in MCF-7, HepG2, SK-N-BE, and HeLa cells transfected with estrogen receptor *α* (ER*α*), whereas E2 was unable to modulate the NGB expression in the ER*α*-devoid HeLa cells. Both transcriptional and extranuclear ER*α* signals were required for the E2-dependent upregulation of NGB in MCF-7 and HepG2 cell lines. E2 stimulation modified NGB intracellular localization, inducing a significant reduction of NGB in the nucleus with a parallel increase of NGB in the mitochondria in both HepG2 and MCF-7 cells. Remarkably, E2 pretreatment did not counteract the H_2_O_2_-induced caspase-3 and poly (ADP-ribose) polymerase 1 (PARP-1) cleavage, as well as Bcl-2 overexpression in MCF-7 and HepG2 cells in which NGB was stably silenced by using shRNA lentiviral particles, highlighting the pivotal role of NGB in E2-induced antiapoptotic pathways in cancer cells. Present results indicate that the E2-induced NGB upregulation in cancer cells could represent a defense mechanism of E2-related cancers rendering them insensitive to oxidative stress. As a whole, these data open new avenues to develop therapeutic strategies against E2-related cancers.

Estrogens, in particular 17*β*-estradiol (E2), regulate a myriad of physiological processes in mammals; thus, it is not surprising that these hormones could influence the incidence of several human diseases including cancer. However, E2 exerts both beneficial and adverse effects on cancer depending on the estrogen receptor subtype (i.e., ER*α* and ER*β*) expressed.^1, 2, 3^ Indeed, ER*α* promotes the proliferation of breast, gynecologic, and endocrine gland cancers. In contrast, ER*β* suppresses the proliferation of tumor cells.^4,5^ The carcinogenic effect of E2 via ER*α* has been highlighted by experiments in ER-knockout mice^6^ and by epidemiological studies on cancer risk in patients who receive female hormone replacement therapy, showing an increased risk to develop breast, gynecologic and endocrine gland cancers. In addition, in E2-sensitive cancer cells (e.g., breast and liver cancers), a progressive increase of ER*α* amount and a parallel decrease of ER*β* expression have been reported.^7^ Remarkably, ER*α* genomic and membrane starting signal transduction pathways cooperatively work to achieve cancer cell proliferation and apoptotic cascade prevention.^1,8, 9, 10^

Neuroglobin (NGB), a 17 kDa heme protein belonging to the globin super-family, was first discovered in neurons of the central and peripheral nervous system.^11^ Recently, we discovered that human NGB is a new contributor of the signal transduction pathways that lead to E2-induced neuronal cell survival. Indeed, E2 enhances NGB protein level particularly in mitochondria, where, after oxidative stress injury, NGB associates with cytochrome *c* impairing its release in the cytosol and the activation of the pro-apoptotic cascade.^12, 13, 14^ Thus, NGB acts as an E2-inducible protein that assures neuronal cell survival in spite of oxidative stress damage.

Besides being a specific nervous system globin,^14, 15, 16^ contradictory evidences of NGB expression in non-nervous normal and tumor tissues have been reported. Indeed, some data suggest that NGB expression is higher in the breast, liver, bladder, and thyroid tumors than in normal tissues;^17,18^ other reports affirm that NGB expression is decreased in hepatoma;^19^ finally, other studies assert that NGB transcript is not detected in matched breast cancer/normal tissue cDNA microarrays.^20^ Thus, in principle, it is possible that the E2-dependent NGB-based protective pathway against oxidative stress could be active also in non-nervous peripheral tissues. In turn, as oxidative stress is a condition frequently occurring in tumor cells,^21^ we sought to determine whether E2-dependent NGB upregulation occurs in non-nervous cancers and is required for cell survival.

Here, we report the existence of an E2-induced NGB upregulation pathway for cell survival in both hepatoma (HepG2) and breast adenocarcinoma (MCF-7) cells, where the E2-pro-oncogenic signals prevail.

## Results

### E2 increases NGB levels in non-nervous cancer cells

**Figure 1:**
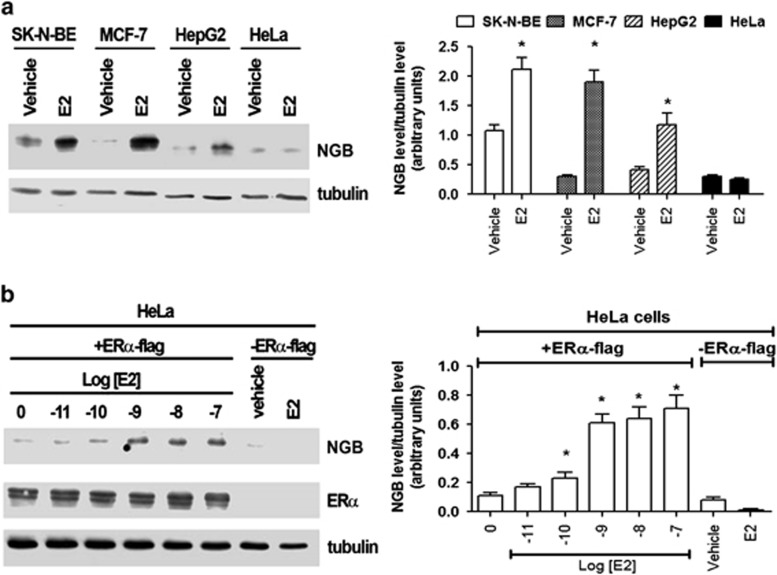
NGB level in cancer cells. (**a**) The effect of E2 (1–10 nM; 24 h stimulation) on NGB protein levels in SK-N-BE, MCF-7, HepG2, and HeLa cells. (**b**) The E2 dose-dependent effect (0.01–100 nM; 24 h stimulation) on NGB level in HeLa cells transfected with pcDNA ER*α*-flag. The amount of protein was normalized by comparison with tubulin levels. The data are typical western blots of three independent experiments and the relative densitometric analyses. Data are means±S.D. of three different experiments. *P*<0.001 was determined with ANOVA followed by Tukey–Kramer post-test *versus* vehicle or 0 (ethanol/PBS 1 : 10, v/v) (*)

According to literature,^22^ neuron-derived cells (i.e., SK-N-BE) contain higher level of NGB with respect to the other non-neuronal cells tested (Figure 1a). E2 (10 nM; 24 h) upregulates NGB level in a similar manner in MCF-7, HepG2, and SK-N-BE cells (Figure 1a). However, the hormone was unable to modulate NGB level in HeLa cells, which are ER-devoid cells (Figure 1a). Remarkably, when HeLa cells were transfected with pcDNA ER*α*-flag, E2 regained its ability to upregulate NGB in a dose-dependent manner, showing an effect already at low E2 concentrations (0.1 nM, Figure 1b).

**Figure 2:**
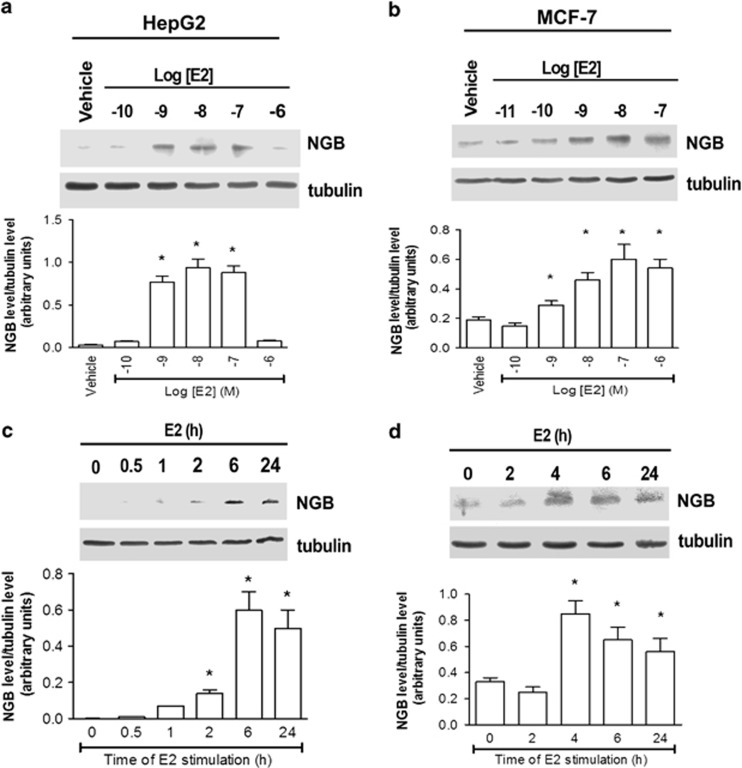
Effect of E2 on NGB protein levels in HepG2 and MCF-7 cells. Western blot analysis of E2 dose-dependent effect (0.1–1000 nM) on NGB levels after 24 h of stimulation in (**a**) HepG2 and (**b**) MCF-7 cells. Time-course analysis of E2 treatment (10 nM) on NGB level in (**c**) HepG2 and (**d**) MCF-7 cells on NGB level. The amount of protein was normalized by comparison with tubulin levels. Top panels are typical western blots of three independent experiments. Bottom panels represent the results of the densitometric analyses

In all the E2-responsive cancer cells tested, E2 induced a dose- and time-dependent increase in NGB levels with a maximum effect at 10 nM (24 h) (Figures 2a and b). The E2 (10 nM) effect on the NGB level began to be significant 2 (HepG2) and 4 h (MCF-7) after the hormone treatment and remained constant until 24 h (Figures 2c and d). According to these results, 10 nM E2 and 24 h of stimulation were used in the following experiments.

### ER involvement in E2-dependent NGB levels

**Figure 3:**
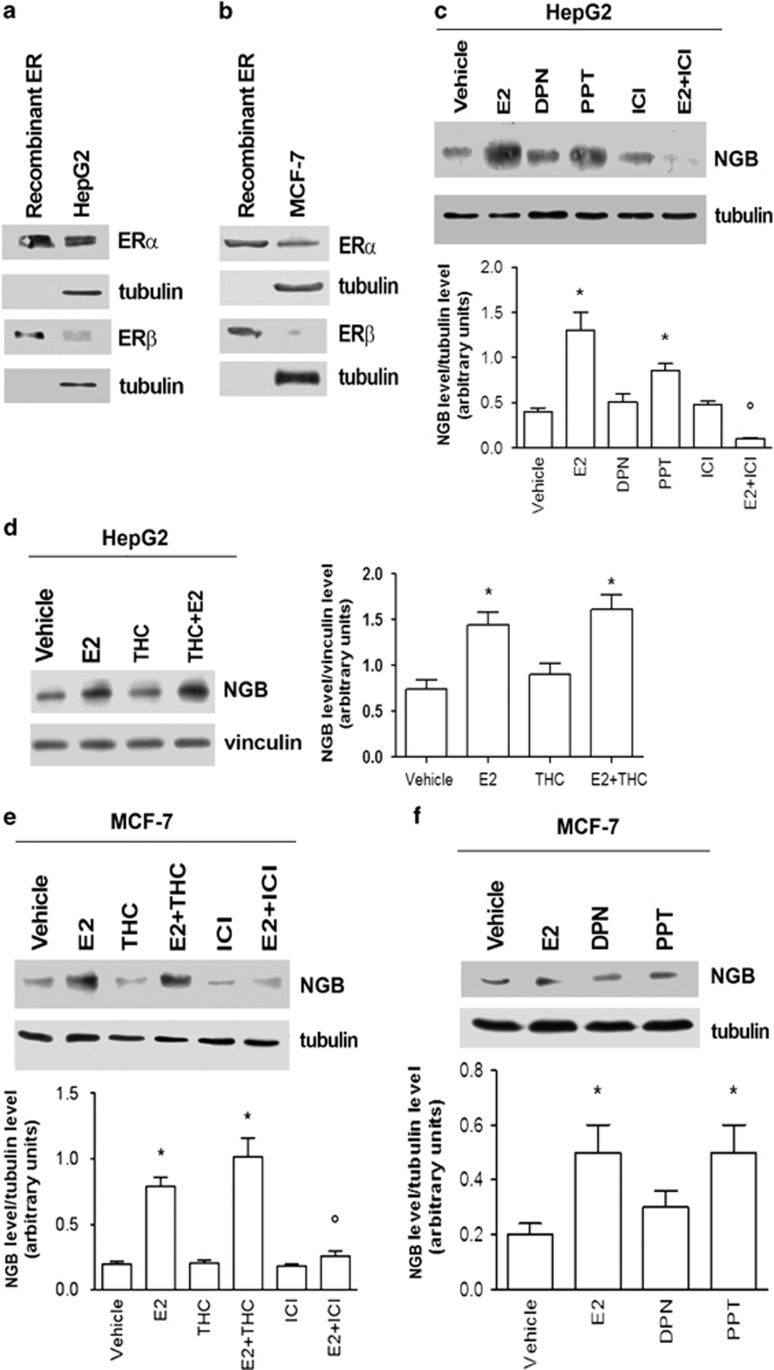
Impact of estrogen receptors on NGB protein expression in HeLa, HepG2, and MCF-7 cells. Western blot of ER*α* and ER*β* levels in non-stimulated cells compared with 5 ng of recombinant proteins in (**a**) HepG2 cells and (**b**) MCF-7 cells. (**c**) Western blot of NGB levels in HepG2 cells stimulated for 24 h with vehicle, E2 (10 nM), ER*β* agonist DPN (10 nM), ER*α* agonist PPT (10 nM) or the ER inhibitor ICI (1 *μ*M) in the absence and presence of E2 (10 nM). (**d**) Western blot of NGB levels in HepG2 cells stimulated for 24 h with vehicle, E2 (10 nM) or ER*β* inhibitor THC (1 *μ*M) in the absence and presence of E2 (10 nM). (**e**) Western blot of NGB levels in MCF-7 cells treated for 24 h with vehicle, E2 (10 nM), ER*β* inhibitor THC (1 *μ*M) or ER inhibitor ICI (1 *μ*M) in the absence and presence of E2 (10 nM). (**f**) Western blot of NGB levels in MCF-7 cells stimulated for 24 h with vehicle, E2 (10 nM), ER*β* agonist DPN (10 nM) or ER*α* agonist PPT (10 nM). The amount of proteins was normalized by comparison with tubulin or vinculin levels. (**a**,**b**) The data are typical western blots of three independent experiments. (**c**–**f**) Top panels are typical western blots, bottom panels represent the results of the densitometric analysis. Data are means±S.D. of three different experiments. *P*<0.001 was determined with ANOVA followed by Tukey–Kramer post-test *versus* vehicle (*) and *versus* E2-treated sample (°)

As expected,^1^ ER*α* is the most abundant receptor subtype expressed in MCF-7 and HepG2 cells, although ER*β* is barely detectable (Figures 3a and b). The pure ER antagonist fulvestrant or ICI 182,780 (ICI) impaired the E2-dependent upregulation of NGB both in MCF-7 (Figure 3c) and HepG2 (Figure 3e) cells. Moreover, the E2 effect in these cells was specifically mediated by ER*α* as deduced by the effect of the specific ER*α* agonist 4,4′,4′′-(4-propyl-(1H)-pyrazole-1,3,5-triyl) trisphenol (PPT) or the specific ER*β* agonist 2,3-bis(4-hydroxyphenyl) propionitrile (DPN) (Figures 3c and f). These data were further confimed by stimulating cells, in the absence and presence of E2 (10 nM), with the ER*β* antagonist (R,R)-5,11-diethyl-5,6,11,12-tetrahydro-2,8-chrysenediol (THC, 1 *μ*M), which did not interfere with the E2 effect on NGB induction in HepG2 (Figure 3d) or MCF-7 (Figure 3e) cells.

### Mechanisms involved in E2-induced NGB upregulation in cancer cell lines

**Figure 4:**
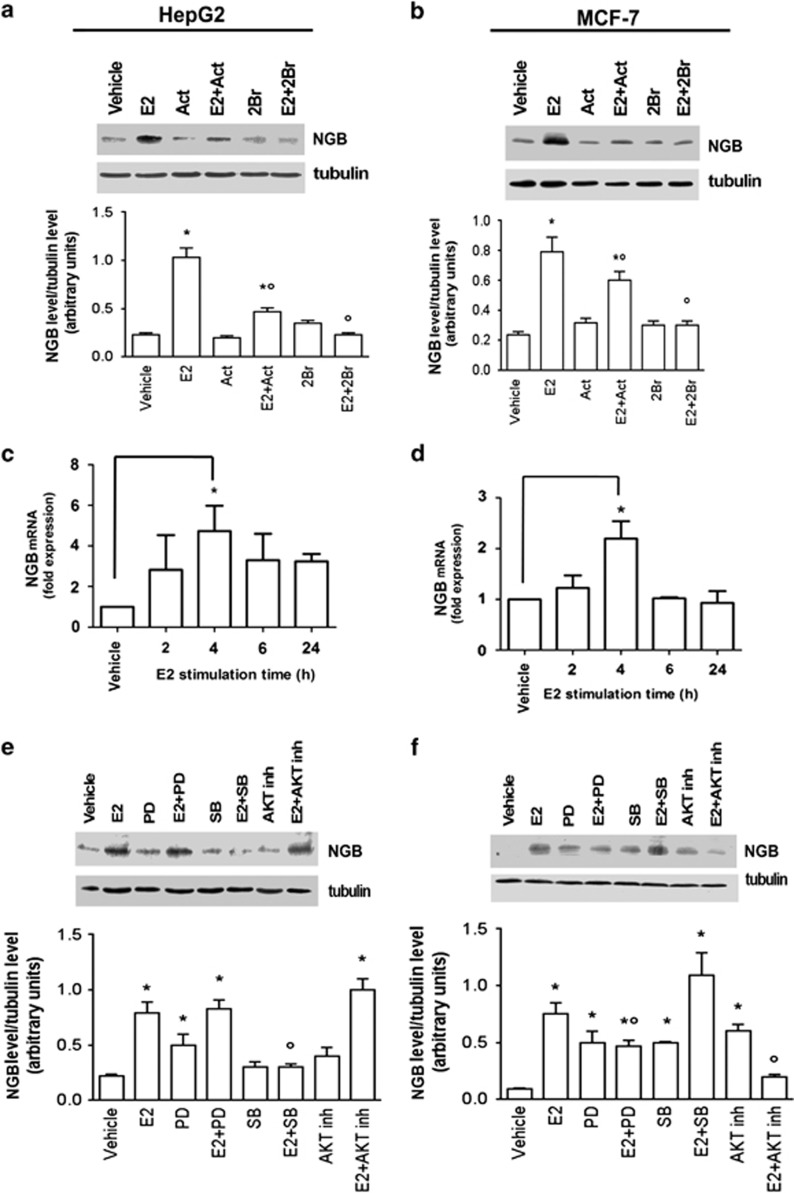
Impact of ER genomic and membrane started signals on NGB protein expression. Analysis of NGB levels in HepG2 (**a**) and MCF-7 (**b**) cells stimulated for 24 h with vehicle, E2 (10 nM) and/or the transcription inhibitor Act (1 *μ*g/ml) and/or the PAT inhibitor 2-Br (10 *μ*M). Analysis of NGB protein levels in (**e**) HepG2 and (**f**) MCF-7 cells treated for 24 h with vehicle, p38 inhibitor SB-203580 (SB; 5 *μ*M) or AKT inhibitor (5 *μ*M) or ERK 1/2 inhibitor PD (10 *μ*M) in the absence or presence of E2 (10 nM). The amount of proteins was normalized by comparison with tubulin levels. Top panels are typical western blots of three independent experiments. Bottom panels represent the results of the densitometric analysis. Data are means±S.D. of three different experiments. *P*<0.001 was determined with ANOVA test *versus* vehicle (*) and *versus* the E2-treated sample (°). Time course of E2 (10 nM) effect on NGB mRNA levels in (**c**) HepG2 and (**d**) MCF-7 cells. The NGB expression is expressed as fold of induction over the vehicle (set to one). Data represent the mean±S.D. of five different experiments. Significant differences (*P*<0.001) were determined by ANOVA followed by the Tukey–Kramer post-test with respect to unstimulated samples (*)

ERs are ligand-activated transcription factors that modulate gene expression by genomic and non-genomic mechanisms.^2,10,23^ In order to evaluate which mechanism(s) is involved in the E2-induced NGB level, HepG2 and MCF-7 cells were stimulated with the specific inhibitor of transcription actinomycin D (Act) or with the inhibitor of the palmitoyl acyltransferase (PAT) 2-bromohexadecanoid acid (2-Br-palmitate or 2-Br) that prevents plasma membrane localization of both receptors.^10,23^ In both cell lines the E2 effect on NGB upregulation was impaired by Act (Figures 4a and b), indicating that the E2-dependent NGB upregulation is transcriptionally modulated as also highlighted by analyzing NGB mRNA levels (Figures 4c and d). In both HepG2 (Figure 4c) and MCF-7 cells (Figure 4d), the E2 treatment induced a significant increase of NGB mRNA levels 4 h after the hormone stimulation, whereas it was not effective at any other time considered. Similarly to the Act treatment, the cell pretreatment with 2-Br also prevented the E2 effect on NGB upregulation, indicating that E2 requires both transcriptional and membrane starting signals to modulate NGB level (Figures 4a and b). This prompted us to evaluate which signal transduction cascade was involved in this E2-dependent effect. As previously reported,^1,24^ E2 (5–10 min, 10 nM) rapidly increased the phosphorylation of p38/mitogen-activated protein kinase (p38/MAPK), protein kinase B (AKT), and extracellular regulated kinase 1/2/MAPK (ERK1/2) in a cell context-dependent manner (Supplementary Figure 1). Notably, the involvement of these kinases in the E2-induced NGB level was also cell context-dependent. Indeed, in HepG2 cells the long-term (i.e., 24 h) E2 effect on NGB was completely blocked only by the p38 inhibitor SB203580 (SB), but not by the AKT and ERK1/2 inhibitors (Figure 4e). On the other hand, AKT and ERK1/2 activation was required for E2 induction of NGB in MCF-7 cells, whereas the p38 inhibitor did not impair E2-induced NGB upregulation (Figure 4f). It should be mentioned that, in MCF-7 cells, the inhibitors of all kinases considered (i.e., MAPK, AKT, and p38) slightly induce the increase of NGB level (Figures 4e and f), whereas only MAPK inhibitor PD98059 (PD) increases NGB level in HepG2 cells.

### E2-dependent intracellular re-allocation of NGB

**Figure 5:**
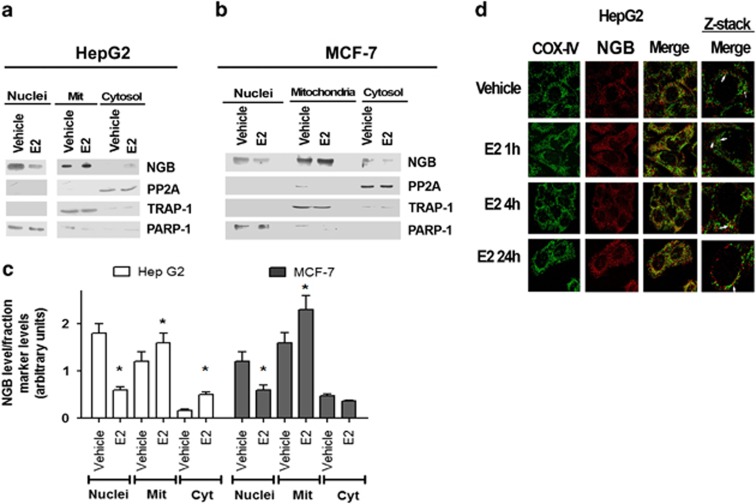
Effect of E2 on the localization of NGB in HepG2 and MCF-7 cells. Western blot of NGB level in nuclear (Nuclei), cytosolic (Cyt), and mitochondrial (Mit) fractions of (**a**) HepG2 and (**b**) MCF-7 cells and related densitometric analyses (c). The purity of fractions was assessed with PARP-1, TRAP1, and PP2A with respect to nucleus, mitochondria, and cytosol, respectively. The amount of proteins was normalized to each fraction marker protein. The figure represents a typical western blot of three independent experiments. Data reported in panel **c** represent the mean±S.D. of three different experiments. Significant differences (*P*<0.001) were determined by ANOVA followed by the Turkey–Kramer post-test with respect to unstimulated samples (*). (**d**) Confocal microscopy analysis of NGB and COX-4 co-immuno-localization. Cells were fixed, permeabilized, and stained with anti-NGB antibody (red) and co-stained with anti-COX4 antibody (green) (original magnification × 63). Merged images by confocal microscopy show NGB distribution in HepG2 cells treated with either vehicle or E2 (10 nM) for 1, 4, and 24 h. White arrows point to examples of NGB and COX-4 co-staining in a single Z-stack plane. Representative images from three different experiments are shown

Recently, we demonstrated that in SK-N-BE cells E2 stimulation induces a localization of NGB at mitochondrial level, where it exerts an antiapoptotic role.^13^ Thus, the analysis of the subcellular localization of NGB was performed also in E2-related non-nervous cancer cells to obtain an inkling of its function in these cells. HepG2 and MCF-7 cells were fractionated into three main compartments (i.e., nuclei, mitochondria, and cytosol) using as fraction purity markers nuclear poly (ADP-ribose) polymerase 1 (PARP-1), mitochondrial TNF receptor-associated protein 1 (TRAP1), and cytosolic protein phosphatase 2A (PP2A) (Figures 5a, b, and c). Upon E2 stimulation (i.e., 10 nM; 24 h), a significant reduction of NGB in the nucleus with the parallel increase of NGB in the mitochondrial fraction in both HepG2 (Figure 5a) and MCF-7 (Figure 5b) cells was observed. However, the E2 treatment led to the increase of the NGB level in the cytosol of HepG2 cells, whereas in MCF-7 cells the amount of cytosolic NGB after E2 stimulation was similar to that observed in the control (Figures 5a and b). A further confirmation that E2 induces NGB re-allocation in mitochondria of HepG2 derives from the confocal microscopy analysis, which indicates that E2 increased the colocalization of NGB with cytochrome *c* oxidase 4 (COX-4) at mitochondrial level beginning 1 h after the stimulation and reaching a maximum 24 h after the hormone treatment (Figure 5d).

### The role played by E2-induced NGB in cancer cells

**Figure 6:**
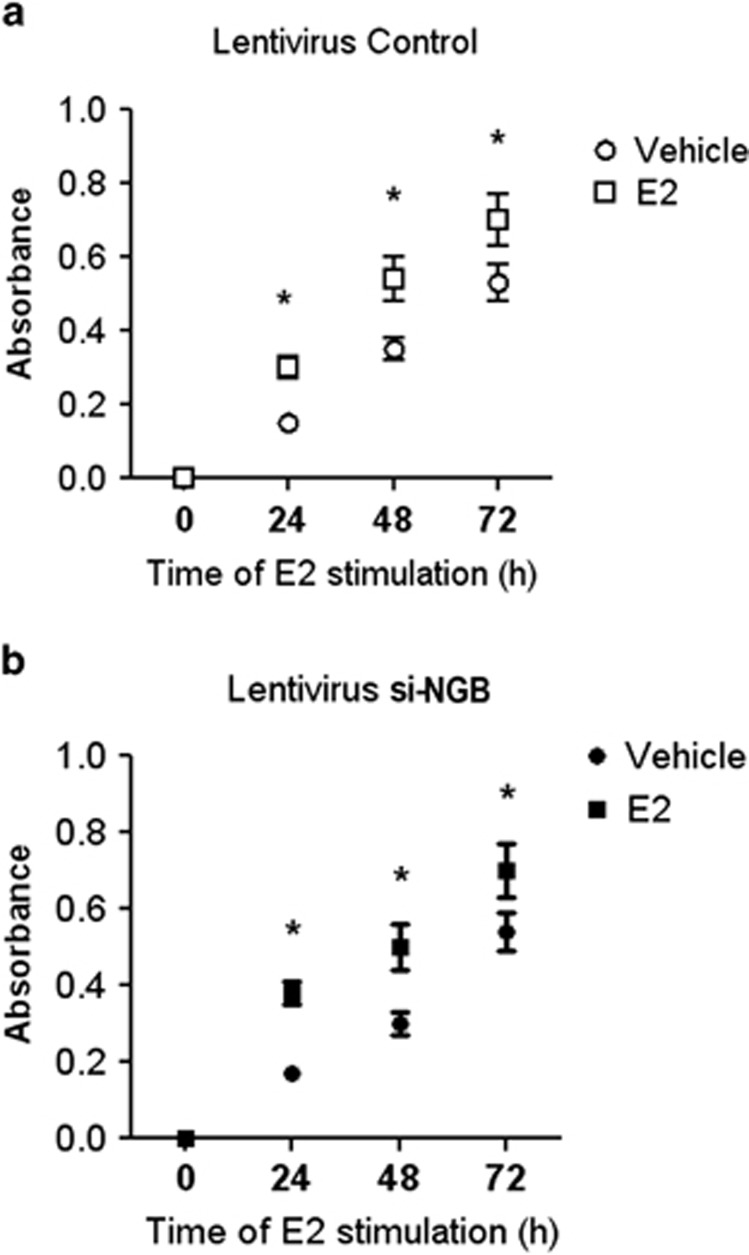
E2 effect on cell proliferation in control and NGB silenced MCF-7 cells. (**a**) Control and (**b**) NGB-silenced MCF-7 cells were stimulated with vehicle or E2 (10 nM). Every 24 h, the XTT labeling mixture was added to the plates and absorbance was measured at 490 nm on an multilabel plate reader. Data are means ± S.D. of three different experiments. *P*<0.001 was determined with ANOVA followed by the Tukey–Kramer post-test *versus* vehicle (*)

The E2–ER*α* complex activates signal transduction pathways (Supplementary Figure 1), which commit cells to cell cycle progression.^1^ Hovewer, E2 treatment (24–72 h) increased the cell number in both control and NGB-silenced MCF-7 cells indicating that NGB is not involved in E2 proliferative effect (Figure 6).

**Figure 7:**
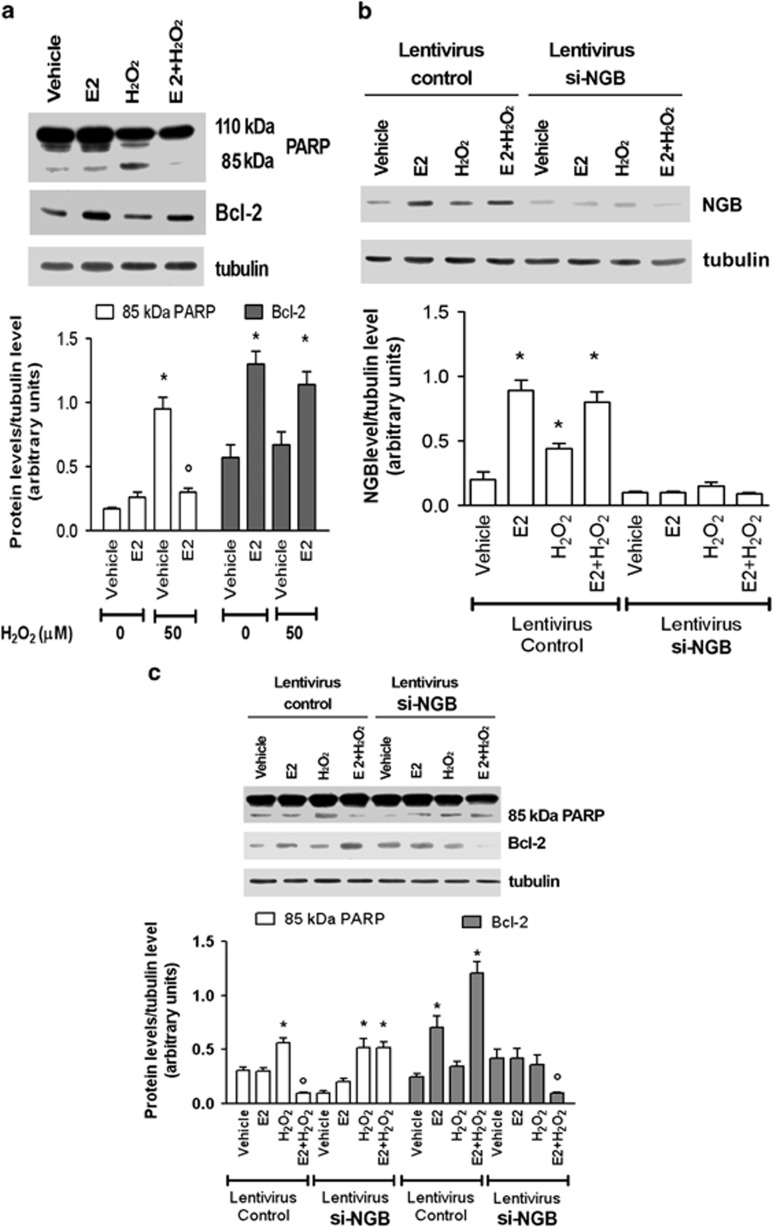
Effect of NGB silencing on caspase-dependent apoptotis in MCF-7 cells. (**a**) Western blot analysis of PARP-1 cleavage and Bcl-2 induction were performed on MCF-7 cells stimulated with either the vehicle or pretreated with E2 (10 nM; 24 h) followed by 24 h treatment with H_2_O_2_ 100 *μ*M. (**b**) Analysis of NGB level in MCF-7 cells infected with either control or NGB shRNA lentiviral particles and stimulated with either the vehicle or pretreated with E2 (10 nM; 24 h) then treated with H_2_O_2_ 100 *μ*M (24 h). (**c**) Western blot analysis of PARP-1 cleavage and Bcl-2 expression were performed in MCF-7 cells infected with either control or NGB shRNA incubated with either vehicle or 100 *μ*M H_2_O_2_ in the presence or absence of 24 h E2 (10 nM) pretreatment. The amount of proteins was normalized to tubulin levels. Top panels are typical western blots of five independent experiments. Bottom panels represent the results of the densitometric analysis. Data are means±S.D. of five different experiments. *P*<0.001 was determined with ANOVA followed by Tukey–Kramer post-test *versus* vehicle (*) and *versus* H_2_O_2_ treated sample (°)

In neurons, the high level of NGB induced by E2 is essential to reset the triggering of apoptosis induced by oxidative stress stimuli.^13,14^ In MCF-7 cells, which lack functional caspase-3 activity,^25^ H_2_O_2_ (100 *μ*M; 24 h) increased the cleavage (band 85 kDa) of PARP-1, the main target of the execution caspase activity, without major effects on the Bcl-2 protein level (Figure 7a). E2 pretreatment (10 nM; 24 h) completely prevented H_2_O_2_-induced PARP-1 cleavage and, in parallel, increased the level of the antiapoptotic protein Bcl-2 (Figure 7a). Similar results were obtained in MCF-7 cells stably transfected with control shRNA lentiviral particles (Figures 7b and c). Notably, H_2_O_2_ (100 *μ*M; 24 h) increased the level of NGB over the control even if to a tlesser extent than E2 (Figure 7b, left panel). On the other hand, MCF-7 cells in which NGB was stably silenced by using shRNA lentiviral particles (Figure 7b), E2 (10 nM; 24 h) pretreatment was not able anymore to counteract the H_2_O_2_- (100 *μ*M; 24 h) induced PARP-1 cleavage and Bcl-2 overexpression (Figure 7c), sustaining the pivotal role of NGB in E2-induced anti-apoptotic pathways. In line with the results obtained in MCF-7 cells, E2 counteracted the H_2_O_2_- (i.e., 50 and 100 *μ*M; 24 h) induced caspase-3 and PARP-1 activation (Supplementary Figure 2a) in HepG2 cells. Similarly to the MCF-7 cells, E2 missed the ability to prevent apoptotic cascade activation in NGB-silenced HepG2 cells (Supplementary Figures 2b and c).

## Discussion

NGB is an oxygen-binding heme protein that has the classical three-on-three globin fold and is widely expressed in the cerebral cortex, hippocampus, thalamus, hypothalamus, cerebellum, and retina of the mammalian brain.^11^ Previous studies have shown that silencing NGB expression decreases neuronal survival after oxidative stress and vice versa.^26^ More recently, it has been demonstrated that NGB is involved in neuroprotection under oxidative stress conditions,^27^ supporting the notion that high levels of NGB could protect neurons from hypoxic/ischemic insults.

Oxidative stress is a condition frequently occurring in fast proliferating neoplastic tissues, and it is now apparent that cancer cells adapt to the imbalanced redox status created by their rapid growth by developing alternative metabolic reactions that render them insensitive to further stress inducers.^21^ This evidence prompted several research groups to study the presence and role of NGB in cancer cells. However, the very low detectable levels of NGB in non-nervous tissues produced only scarce and controversial data.^17, 18, 19, 20^

The main aim of this paper was to evaluate the possibility that NGB is upregulated by E2 also in cancer cells, where NGB upregulation would increase cell survival after oxidative stress, acting as a mediator of E2 pro-oncogenic effects.

The results reported here indicate that the NGB basal level was barely recognizable in MCF-7 and HepG2 cells in comparison with neuronal-derived cells SK-N-BE, according to that reported by other authors in tumor tissues.^20,27^ Therefore, also in cancer cells NGB should be considered as an E2-inducible protein apart from the ER-devoid HeLa cells. In fact, E2 acquires the ability to increase NGB levels in HeLa cells only when ER*α* levels were rescued by transfecting cells with the ER*α*-encoding plasmid. All together, these results show, for the first time, that E2 is able to modulate NGB levels in E2-related non-nervous cancer cells through ER*α*.

In ER*α*-containing HepG2 and MCF-7 cells, both nuclear (transcriptional) and membrane starting (extranuclear) ER*α* action mechanisms are required. Indeed, the transcription inhibitor Act completely prevents the E2-dependent increase of NGB levels and E2 upregulates NGB mRNA levels in both cell types. Although NGB promoter does not contain any canonical estrogen-responsive element (ERE) consensus sequence, it includes several responsive elements for other transcription factors (i.e., AP-1 and Sp-1),^28^ suggesting that a mechanism of indirect transcription could be involved in the genomic regulation of NGB expression. Accordingly, E2 induces the activation of the three common ER signaling pathways (i.e., ERK, PI3K/AKT, and p38/MAPK).^10^ However, they are differently involved in E2-induced NGB upregulation. In fact, ERK and AKT activation are necessary in MCF-7 cells, whereas the rapid (5 min) and transient (10 min) p38 activation is specifically required in HepG2 cells. Intriguingly, the inhibitors of all kinases considered (i.e., MAPK, AKT, and p38) slightly increases NGB level in MCF-7 cells, whereas only MAPK inhibitor PD increases NGB level in HepG2 cells, suggesting the existence of a context-specific cross-talk between the kinases in regulating NGB levels both at the baseline and upon E2 stimulation. More experiments are needed to better clarify the signal pathways involved in the regulation of NGB levels in cancer cells. This issue is under investigation in our lab. However, the different ER*α* splice variants expressed by HepG2 and MCF-7 cells may account for the different rapid membrane starting signals required for the E2-dependent NGB modulation. Indeed, in MCF-7 cells, beyond the functional ER*α* wild-type isoform (66 kDa), other splice variants are expressed, including the ER*α*-36, which is stably localized at the plasma membrane.^29^ On the other hand, HepG2 cells contain only a variant of ER*α* full length (66 kDa), which is unable to directly transactivate ERE-containing reported genes, but mediates the E2-induced indirect transcription of *cyclin D1* gene.^30^ The presence of these ER*α* splice variants sustains that E2 regulates NGB expression mainly by ER*α*-activated rapid mechanisms that in turn trigger indirect nuclear events to modulate the NGB expression. These findings lead to a change in the dogma, which considers NGB as a specific nervous system globin, suggesting a new perspective of NGB as a pivotal player in the E2 signal pathway in several hormone target cell lines.

High oxidative stress represents a hallmark of many cancers^31^ and could drive cancer cells to apoptosis.^32^ However, cancer cells undergo metabolic and transcriptional adaptations against stress agents, allowing cancer cells to survive even when the redox status is imbalanced.^21^ NGB could act as a compensatory protein in E2-related cancers as reported in neurons.^27^ The results reported here confirm that H_2_O_2_ renders cells more prone to the pro-apoptotic cascade activation in both HepG2 and MCF-7 cells. In addition, H_2_O_2_ treatment slightly increases NGB level in MCF-7 cells (91±10.2% over the control); however, the protein level is not sufficient to prevent apoptosis. *In vitro*, cell protection against apoptosis has been postulated to be achieved when the NGB/mitochondrial cytochrome *c* ratio is at least 3/1.^33^ In line with these *in vitro* results, E2 increases NGB level of 274±20.6% over the control. This high NGB level seems to be essential for the E2 anti-apoptotic effects against the H_2_O_2_ toxicity in ER*α*-expressing cancer cells. In addition, in MCF-7 cells, NGB is also required for the E2-dependent increase of Bcl-2 expression level. Recently, it has been suggested that the overexpression of NGB in HepG2 cells, after transfection with an encoding plasmid, displays a tumor suppressor function.^19^ Although it is difficult to compare our cells with NGB-transfected ones, the highest level of NGB obtained after transfection could likely interfere with signal transduction pathways (i.e., ERK activation) that drive cancer cells to proliferation.^19^ In addition, the evidence that the inhibitors of the fundamental cellular signaling cascade regulating tumor development, growth, proliferation, and metastasis^34^ slightly increase NGB level (Figures 4e and f) strongly sustains the idea that reduction of cancer cell proliferation could activate compensatory mechanism(s) devoted to cancer cell survival. Although more work is needed to sustain this idea, our data strongly support the view that the physiological induction of NGB level triggered by E2 has a compensatory role against oxidative stress. Moreover, NGB silencing in MCF-7 cells does not interfere with the E2-dependent increase of cell proliferation, highlighting that the NGB upregulation induced by E2 is specifically involved in protecting cancer cells against stress-induced apoptosis.

Among the different proposed mechanisms for NGB antiapoptotic role, the direct involvement of NGB in the intrinsic apoptotic pathway at mitochondrial level has been identified in neurons.^13^ As reported here, also in non-nervous cancer cells, as well as in neuroblastoma cells, E2 treatment significantly increases the amount of NGB in the mitochondrial compartment, strengthening the idea that NGB could represent the intracellular mediator of the E2 antiapoptotic effect in ER*α*-encoding cancer cells, acting directly in the mitochondria, where the core of the intrinsic apoptotic pathway initiates.

In conclusion, the present results indicate that E2-induced NGB upregulation in cancer cells could represent a defense mechanism of E2-related cancers against oxidative stress. NGB, as a defense protein, could even render cancer cells insensitive to other stress inducers, such as chemotherapy and radiation. Although further studies are needed to better dissect the role of NGB in E2 cancerogenesis, the present data clarify the functional role played by NGB in tumor cells opening new avenues to develop therapeutic strategies against E2-related cancers.

## Materials and Methods

### Reagents

E2, Act, Pen-Strep solution, H_2_O_2_, RPMI-1640 media without phenol red, Dulbecco's modified Eagle's medium without phenol red, charcoal-stripped fetal calf serum, the PAT inhibitor 2-Br, protease inhibitor cocktail, bovine serum albumin fraction V (BSA), and mouse monoclonal anti-FlagM2 antibody were purchased from Sigma-Aldrich (St. Louis, MO, USA). Optimem, Hank's buffer salt solution (1 × ), and GlutaMAX-I were purchased from Gibco-BRL (Gaithersburg, MD, USA). The p38 inhibitor SB, the AKT inhibitor, and the MAPK cascade inhibitor PD were obtained from Calbiochem (San Diego, CA, USA). The E2 antagonist ICI, ER*α*-selective agonist PPT, ER*β*-selective agonist DPN, and ER*β*-selective antagonist THC were obtained from Tocris (Ballwin, MO, USA). Bradford protein assay was obtained from Bio-Rad Laboratories (Hercules, CA, USA). Human recombinant ER*α* and ER*β* were obtained by Invitrogen (Carlsbad, CA, USA). NGB shRNA lentiviral particles, control shRNA lentiviral particles, anti-phospho-ERK1/2, anti-AKT, anti-ER*α* (MC-20), anti-ER*β* (H-150), anti-caspase-3, anti-PARP-1, anti-PP2A, anti-ERK1/2, anti-Hsp75 (TRAP1), anti-NGB, and anti-Bcl-2 antibodies were obtained from Santa Cruz Biotechnology (Santa Cruz, CA, USA). Polyclonal anti-phospho-AKT, anti-phospho-p38, and anti-p38 antibodies were purchased from New England Biolabs (Beverly, MA, USA). The anti-*α*-tubulin was purchased from Sigma-Aldrich. Anti-COX-4 antibody was purchased from Clontech Laboratories (Mountain View, CA, USA). The chemiluminescence reagent for western blot super power ECL was obtained from Bio-Rad (Milan, Italy). Cell proliferation Kit II (XTT) was purchased from Roche (Basel, Switzerland). All the other products were from Sigma-Aldrich. Analytical or reagent grade products were used without further purification.

Cell growth and proliferation of SK-N-BE(2)-C, MCF-7, HepG2, and HeLa cell lines (ATCC, LGC Standards S.r.l., Milan, Italy) were used at passage 4–8 and were grown as previously described.^1,12,35^ Cell line authentication were periodically performed by amplification of multiple STR loci by BMR Genomics srl (Padua, Italy). Lentiviral-infected MCF-7 and HepG2 cell lines were routinely grown in media containing puromycin (0.5 mg/ml and 3 mg/ml, respectively). Cells were simultaneously treated with the vehicle used to dissolve all drugs (ethanol/PBS 1 : 10, v/v), and/or E2 (0.01–1000 nM) or PPT (0.01–100 nM) or DPN (0.01–100 nM) and/or H_2_O_2_ (100 *μ*M). When indicated, the PAT inhibitor 2-Br (10 *μ*M), the anti-estrogen ICI (1 *μ*M), the ER*β* inhibitor THC (1 *μ*M), the AKT inhibitor (1 *μ*M), the p38 inhibitor SB (5 *μ*M), the ERK1/2 inhibitor PD (1 *μ*M), and the transcription inhibitor Act (1 *μ*g/ml) were added 30 min before E2-MCF-7 cell proliferation was assessed by the XTT assay (Roche) according to the manufacturer's instructions. The microplates were read at 490 nm on a multilabel plate reader (VICTOR X3 Multilabel Plate Reader, PerkinElmer, Waltham, MA, USA) to measure the absorbance.

Transfection of Flag-ER*α* plasmid, the reporter plasmid 3x ERE TATA, the pcDNA Flag 3.1 C, and pcDNA Flag-ER*α* were previously described.^36^ HeLa cells were grown to ~70% confluence and then transfected with pcDNA-Flag-ER*α* plasmid using lipofectamine (Invitrogen) according to the manufacturer's instructions. Six hours after transfection the medium was changed, and after 24 h the cells were treated with either vehicle or E2 (10 nM).

### Cellular and biochemical assays

Protein extraction, western blot assay, and cell fractionation were performed as previously described.^13^ Briefly, cell fractionation was performed using ApoAlert Cell Fractionation kit (Clontech Laboratories) according to the manufacturer's instructions. After stimulation, cells were harvested with trypsin (1%, v/v), suspended with complete medium, and centrifuged at 600 × *g* for 5 min. The pellet was suspended in a fractionation buffer mix containing DTT (1 mM) and homogenized in a Dounce tissue grinder. The homogenate was centrifuged at 700 × *g* for 10 min. The pellet was suspended in the sample buffer containing 0.125 M Tris, pH 6.8 and 10% (w/v) SDS (nuclear fraction); the supernatant was centrifuged again at 10 000 × *g* for 25 min. The supernatant was collected (cytosolic fraction) and the pellet was suspended in a fractionation buffer mix (mitochondrial fraction). The protein concentration of each fraction was determined using Bradford protein assay. The lysate of each fraction was then processed for western blot or used for immunoprecipitation assay.

After treatments, cells were lysed and solubilized as previously described.^13^ Total proteins were quantified using the Bradford protein assay. Solubilized proteins (20 *μ*g) were resolved by 7 or 15% SDS-PAGE at 100 V for 1 h at 25 °C and then electrophoretically transferred to nitrocellulose by for 7 min at 25 V by using Trans Blot Turbo Transfer System (Bio-Rad). The nitrocellulose was treated with 3% (w/v) BSA in 138 mM NaCl, 25 mM Tris, pH 8.0, and 0.1% (w/v) Tween-20 at 25 °C for 1 h and then probed overnight at 4 °C with anti-NGB (final dilution 1 : 1000), anti-ER*α* MC-20 (final dilution 1 : 500), anti-ER*β* H-150 (final dilution 1 : 3000), anti-caspase-3 (final dilution 1 : 1000), anti-PARP (final dilution 1 : 500), anti-phospho-ERK1/2 (final dilution 1 : 200), anti-phospho-AKT (final dilution 1 : 1000), anti-phospho-p38 (final dilution 1 : 1000), anti-COX-4 (final dilution 1 : 1000) or anti-PP2A (final dilution 1 : 1000), anti-Bcl-2 (final dilution 1 : 500), anti-TRAP1 (final dilution 1 : 1000), and anti-Bax (final dilution 1 : 500) antibodies. The nitrocellulose was stripped by Restore Western Blot Stripping Buffer (Pierce Chemical, Rockford, IL, USA) for 10 min at room temperature and then probed with anti-*α*-tubulin (final dilution 1 : 5000) or anti-vinculin (final diluition 1 : 5000) to normalize protein content. Moreover, the nitrocellulose incubated with anti-phospho-ERK1/2, anti-phospho-AKT or anti-phospho-p38 was stripped and probed with anti-ERK1/2 (final dilution 1 : 200), anti-AKT (final dilution 1 : 100) and anti-p38 (final dilution 1 : 1000) antibodies, respectively. To evidence ER*α* and ER*β* levels, electrophoresis was performed in the presence of 5 ng of recombinant proteins. Antibody reaction was visualized with ECL chemiluminescence. Densitometric analyses were performed by ImageJ software for Microsoft Windows (NIH, Bethesda, MD, USA). The densitometry quantification of protein was normalized to tubulin or vinculin.

### Confocal microscopy analysis

HepG2 cells were stained with anti-NGB (1 : 200) and anti-COX-4 (1 : 200) antibodies, respectively. Cells were processed in chamber slides and rinsed with PBS, pH 7.4, followed by fixation in formaldehyde 4% (v/v) for 1 h, and permeabilization with cold acetone 95% for 3 min. Cells were rinsed in PBS and saturated with BSA 2% (w/v) for 1 h and then incubated with the primary antibody at 4 °C overnight (anti-NGB) or 1 h at room temperature (anti-FlagM2). Cells were rinsed three times in PBS for 5 min and incubated with Alexa Fluor 488 donkey anti-mouse secondary antibodies (Invitrogen) (1 : 400) and 578 donkey anti-rabbit secondary antibodies (Invitrogen) (1 : 400). The slides were cover-slipped using Prolong Gold anti-fade reagent. Confocal analysis ( × 63 magnification) was performed using LCS (Leica Microsystems, Milan, Italy).

### Quantitative real-time PCR

The sequences for gene-specific forward and reverse primers were designed using the OligoPerfect Designer software program (Invitrogen). The following primers were used: 5'-GTCTCTCCTCGCCTGAGTTC-3' (forward) and 5'-GACTCACCCACTGTCGAGAA-3' (reverse) for human NGB, and 5′-CGAGATCCCTCCAAAATCAA-3′ (forward) and 5′-TGTGGTCATGAGTCCTTCCA-3′ (reverse) for human GAPDH. Total RNA was extracted from cells using TRIzol Reagent (Invitrogen) according to the manufacturer's instructions. To determine NGB expression levels, cDNA synthesis and qPCR were performed using the GoTaq two-step RT-qPCR system (Promega, Fitchburg, WI, USA) in an ABI Prism 7900HT Sequence Detection System (Applied Biosystems, Foster City, CA, USA) according to the manufacturer's instructions. Each sample was tested in triplicate and the experiment was repeated twice. All primers used were optimized for real-time amplification in a standard curve amplification (>98% for each pair of primers); moreover, the production of a single amplicon in a melting curve assay was verified. Results were normalized to the expression of GAPDH mRNA. The relative level for each gene reported in arbitrary units was calculated by using the 2-ΔΔCt method.^36^

shRNA lentiviral particle transduction was performed using control shRNA lentiviral particles (Santa Cruz sc-108080) and NGB shRNA lentiviral particles (Santa Cruz sc-42081-v) according to the manufacturer's instructions. Cells were plated in a 12-well plate for 24 h before the viral infection. Cells were grown to ~50% confluence and then infected. Growth media were removed and replaced with 1 ml of a mixture of complete medium with Polybrene (Santa Cruz sc-134220) at a final concentration of 5 *μ*g/ml; then, shRNA lentiviral larticles were added to the culture. After 24 h of incubation, the culture medium was removed and replaced with 1 ml of complete medium without Polybrene. After overnight incubation, cells were splited 1 : 3 and maintained in incubation for 24 h in the complete medium. Finally, pooled stable clones expressing the shRNA were selected by incubating cells with a mixture of complete medium with puromycin. Next, the medium was replaced with fresh puromycin-containing medium every 3 to 4 days until resistant colonies were identified. Colonies were expanded and assayed for stable shRNA expression.

Statistical analysis was performed by using ANOVA followed by Tukey–Kramer post-test with the GraphPad InStat3 software system for Windows (La Jolla, CA, USA). In all cases, *P*<0.05 was considered significant.

## Abbreviations

2-Br: 2-bromohexadecanoid acid (also named 2-Br-palmitate)
Act: actinomycin D
AKT: protein kinase B
BSA: bovine serum albumin fraction V
COX-4: cytochrome *c* oxidase-4
DPN: 2,3-bis(4-hydroxyphenyl) propionitrile
E2: 17*β*-estradiol
ER*α*: estrogen receptor *α*
ER*β*: estrogen receptor *β*
ERK1/2: extracellular regulated kinase 1/2
H-150: anti-ER*β* antibody
ICI 182,780: fulvestrant (also named ICI)
MAPK: mitogen-activated protein kinase
MC-20: anti-ER*α* antibody
NGB: human neuroglobin
PARP-1: poly (ADP-ribose) polymerase 1
PAT: palmitoyl acyltransferase
PD98059: MAPK inhibitor
PP2A: protein phosphatase 2 A
PPT: 4,4',4''-(4-propyl-(1H)-pyrazole-1,3,5-triyl) trisphenol
SB203580: p38 inhibitor
THC: (R,R)-5,11-diethyl-5,6,11,12-tetrahydro-2,8-chrysenediol
TRAP1: TNF receptor-associated protein 1
XTT: 2,3-bis-(2-methoxy-4-nitro-5-sulfophenyl)-2H-tetrazolium-5-carboxanilide, Cell proliferation Kit

## References

[bib1] 1Acconcia F, Totta P, Ogawa S, Cardillo I, Inoue S, Leone S et al. Survival versus apoptotic 17*β*-estradiol effect: role of ER*α* and ER*β* activated non-genomic signaling. J Cell Physiol 2005; 203: 193–201. 15389627 10.1002/jcp.20219

[bib2] 2Ascenzi P, Bocedi A, Marino M. Structure-function relationship of estrogen receptor *α* and *β*: impact on human health. Mol Aspects Med 2006; 27: 299–402. 16914190 10.1016/j.mam.2006.07.001

[bib3] 3Burns KA, Korach KS. Estrogen receptors and human disease: an update. Arch Toxicol 2012; 86: 1491–1504. 22648069 10.1007/s00204-012-0868-5 PMC4782145

[bib4] 4Marino M. Xenoestrogens challenge 17*β*-estradiol protective effects in colon cancer. World J Gastrointest Oncol 2014; 6: 67–73. 24653796 10.4251/wjgo.v6.i3.67 PMC3955780

[bib5] 5Nilsson S, Gustafsson JÅ. Estrogen receptors: therapies targeted to receptor subtypes. Clin Pharmacol Ther 2011; 89: 44–55. 21124311 10.1038/clpt.2010.226

[bib6] 6Dupont S, Krust A, Gansmuller A, Dierich A, Chambon P, Mark M. Effect of single and compound knockouts of estrogen receptors *α* (ER*α*) and *β* (ER*β*) on mouse reproductive phenotypes. Development 2000; 127: 4277–4291. 10976058 10.1242/dev.127.19.4277

[bib7] 7Chen GG, Zeng Q, Tse GM. Estrogen and its receptors in cancer. Med Res Rev 2008; 28: 954–974. 18642351 10.1002/med.20131

[bib8] 8Marquez DC, Chen HW, Curran EM, Welshons WV, Pietras RJ. Estrogen receptors in membrane lipid rafts and signal transduction in breast cancer. Mol Cell Endocrinol 2006; 246: 91–100. 16388889 10.1016/j.mce.2005.11.020

[bib9] 9Marino M, Ascenzi P. Membrane association of estrogen receptor *α* and *β* influences 17*β*-estradiol-mediated cancer cell proliferation. Steroids 2008; 73: 853–858. 18206197 10.1016/j.steroids.2007.12.003

[bib10] 10Acconcia F, Marino M. The effects of 17*β*-estradiol in cancer are mediated by estrogen receptor signaling at the plasma membrane. Front Physiol 2011; 2: 30. 21747767 10.3389/fphys.2011.00030 PMC3129035

[bib11] 11Ascenzi P, Gustincich S, Marino M. Mammalian nerve globins in search of functions. IUBMB Life 2014; 66: 268–276. 24753139 10.1002/iub.1267

[bib12] 12De Marinis E, Ascenzi P, Pellegrini M, Galluzzo P, Bulzomi P, Arevalo MA et al. 17*β*-estradiol - a new modulator of neuroglobin levels in neurons: role in neuroprotection against H_2_O_2_-induced toxicity. Neurosignals 2010; 18: 223–235. 21335947 10.1159/000323906

[bib13] 13De Marinis E, Fiocchetti M, Acconcia F, Ascenzi P, Marino M. Neuroglobin upregulation induced by 17*β*-estradiol sequesters cytocrome *c* in the mitochondria preventing H_2_O_2_-induced apoptosis of neuroblastoma cells. Cell Death Dis 2013; 4: e508. 23429294 10.1038/cddis.2013.30 PMC3734830

[bib14] 14Fiocchetti M, De Marinis E, Ascenzi P, Marino M. Neuroglobin and neuronal cell survival. Biochim Biophys Acta 2013; 1834: 1744–1749. 23357651 10.1016/j.bbapap.2013.01.015

[bib15] 15Khan AA, Wang Y, Sun Y, Mao XO, Xie L, Miles E et al. Neuroglobin-overexpressing transgenic mice are resistant to cerebral and myocardial ischemia. Proc Natl Acad Sci USA 2006; 103: 17944–17948. 17098866 10.1073/pnas.0607497103 PMC1693852

[bib16] 16Jin K, Mao XO, Xie L, Khan AA, Greenberg DA. Neuroglobin protects against nitric oxide toxicity. Neurosci Lett 2008; 430: 135–137. 18035490 10.1016/j.neulet.2007.10.031 PMC2265792

[bib17] 17Emara M, Turner AR, Allalunis-Turner J. Hypoxic regulation of cytoglobin and neuroglobin expression in human normal and tumor tissues. Cancer Cell Int 2010; 10: 33. 20828399 10.1186/1475-2867-10-33 PMC2945342

[bib18] 18Oleksiewicz U, Daskoulidou N, Liloglou T, Tasopoulou K, Bryan J, Gosney JR et al. Neuroglobin and myoglobin in non-small cell lung cancer: expression, regulation and prognosis. Lung Cancer 2011; 74: 411–418. 21640426 10.1016/j.lungcan.2011.05.001

[bib19] 19Zhang J, Lan SJ, Liu QR, Liu JM, Chen XQ. Neuroglobin, a novel intracellular hexa-coordinated globin, functions as a tumor suppressor in hepatocellular carcinoma via Raf/MAPK/Erk. Mol Pharmacol 2013; 83: 1109–1119. 23478801 10.1124/mol.112.083634

[bib20] 20Gorr TA, Wichmann D, Pilarsky C, Theurillat JP, Fabrizius A, Laufs T et al. Old proteins - new locations: myoglobin, haemoglobin, neuroglobin and cytoglobin in solid tumours and cancer cells. Acta Physiol (Oxf) 2010; 202: 563–581. 20958924 10.1111/j.1748-1716.2010.02205.x

[bib21] 21Gorrini C, Harris IS, Mak TW. Modulation of oxidative stress as an anticancer strategy. Nature Rev-Drug Discov 2013; 12: 931–947. 24287781 10.1038/nrd4002

[bib22] 22Burmester T, Hankeln T. What is the function of neuroglobin?. J Exp Biol 2009; 10: 1423–1428. 10.1242/jeb.000729 19411534

[bib23] 23Marino M, Galluzzo P, Ascenzi P. Estrogen signaling multiple pathways to impact gene transcription. Curr Genomics 2006; 7: 497–508. 18369406 10.2174/138920206779315737 PMC2269003

[bib24] 24Galluzzo P, Caiazza F, Moreno S, Marino M. Role of ER*β* palmitoylation in the inhibition of human colon cancer cell proliferation. Endocr Relat Cancer 2007; 14: 153–167. 17395984 10.1677/ERC-06-0020

[bib25] 25Essmann F, Engels IH, Totzke G, Schulze-Osthoff K, Janicke RU. Apoptosis resistance of MCF-7 breast carcinoma cells to ionizing radiation is independent of p53 and cell cycle control but caused by the lack of caspase-3 and a caffeine-inhibitable event. Cancer Res 2004; 64: 7065–7072. 15466201 10.1158/0008-5472.CAN-04-1082

[bib26] 26Li D, Chen XQ, Li WJ, Yang YH, Wang JZ, Yu AC. Cytoglobin up-regulated by hydrogen peroxide plays a protective role in oxidative stress. Neurochem Res 2007; 32: 1375–1380. 17476593 10.1007/s11064-007-9317-x

[bib27] 27Watanabe S, Takahashi N, Uchida H, Wakasugi K. Human neuroglobin functions as an oxidative stress-responsive sensor for neuroprotection. J Biol Chem 2012; 287: 30128–30138. 22787149 10.1074/jbc.M112.373381 PMC3436268

[bib28] 28Cutrupi S, Ferrero G, Reineri S, Cordero F, De Bortoli M. Genomic lens on neuroglobin transcription. IUBMB Life 2014; 66: 46–51. 24395659 10.1002/iub.1235

[bib29] 29Shi L, Dong B, Li Z, Lu Y, Ouyang T, Li J et al. Expression of ER-*α*36, a novel variant of estrogen receptor *α*, and resistance to tamoxifen treatment in breast cancer. J Clin Oncol 2009; 27: 3423–3429. 19487384 10.1200/JCO.2008.17.2254 PMC2717750

[bib30] 30Marino M, Acconcia F, Bresciani F, Weisz A, Trentalance A. Distinct nongenomic signal transduction pathways controlled by 17beta-estradiol regulate DNA synthesis and cyclin D(1) gene transcription in HepG2 cells. Mol Biol Cell 2002; 13: 3720–3729. 12388769 10.1091/mbc.E02-03-0153 PMC129978

[bib31] 31Brahimi-Horn MC, Chiche J, Pouyssegur J. Hypoxia and cancer. J Mol Med (Berl) 2007; 85: 1301–1307. 18026916 10.1007/s00109-007-0281-3

[bib32] 32Wang X, Dykens JA, Perez E, Liu R, Yang S, Covey DF et al. Neuroprotective effects of 17*β*-estradiol and nonfeminizing estrogens against H_2_O_2_ toxicity in human neuroblastoma SK-N-SH cells. Mol Pharmacol 2006; 70: 395–404. 16614138 10.1124/mol.106.022384

[bib33] 33Raychaudhuri S, Skommer J, Henty K, Birch N, Brittain T. Neuroglobin protects nerve cells from apoptosis by inhibiting the intrinsic pathway of cell death. Apoptosis 2010; 15: 401–411. 20091232 10.1007/s10495-009-0436-5 PMC2845893

[bib34] 34Polivka J Jr, Janku F. Molecular targets for cancer therapy in the PI3K/AKT/mTOR pathway. Pharmacol Ther 2014; 142: 164–175. 24333502 10.1016/j.pharmthera.2013.12.004

[bib35] 35Bulzomi P, Bolli A, Galluzzo P, Acconcia F, Ascenzi P, Marino M. The naringenin-induced proapoptotic effect in breast cancer cell lines holds out against a high bisphenol a background. IUBMB Life 2012; 64: 690–696. 22692793 10.1002/iub.1049

[bib36] 36La Rosa P, Pesiri V, Marino M, Acconcia F. 17*β*-Estradiol- induced cell proliferation requires estrogen receptor (ER) *α* monoubiquitination. Cell Signal 2011; 23: 1128–1135. 21356307 10.1016/j.cellsig.2011.02.006

