# Supplementary figures and images for: Neuroglobin, a pro-survival player in estrogen receptor α-positive cancer cells
Source: Cell Death Dis. 2014 Oct 9;5(10):e1449–. doi: 10.1038/cddis.2014.418 (PMC4237245; doi:10.1038/cddis.2014.418)

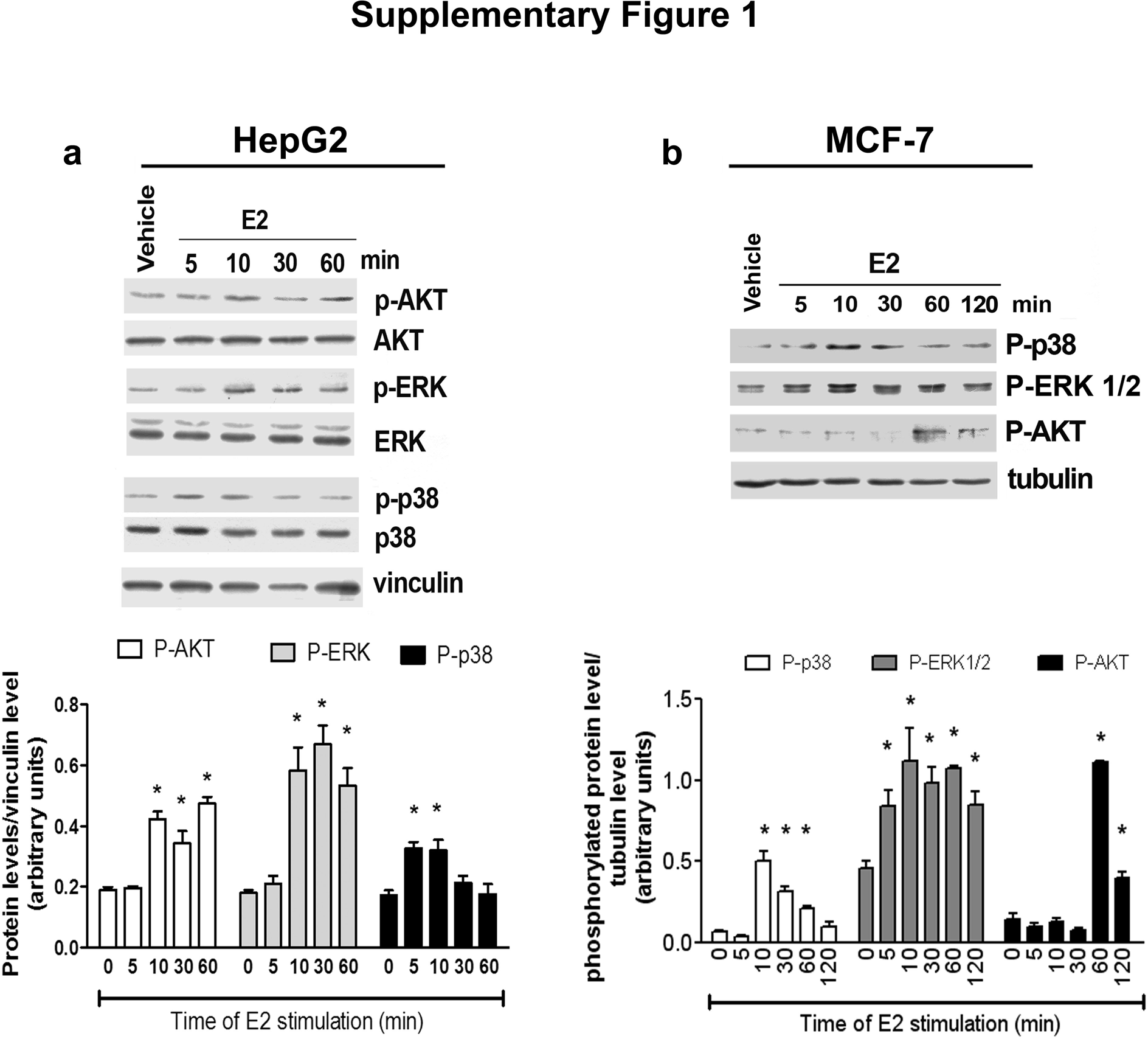

Supplement: Supplementary Figure 1 [file cddis2014418x1.tif]

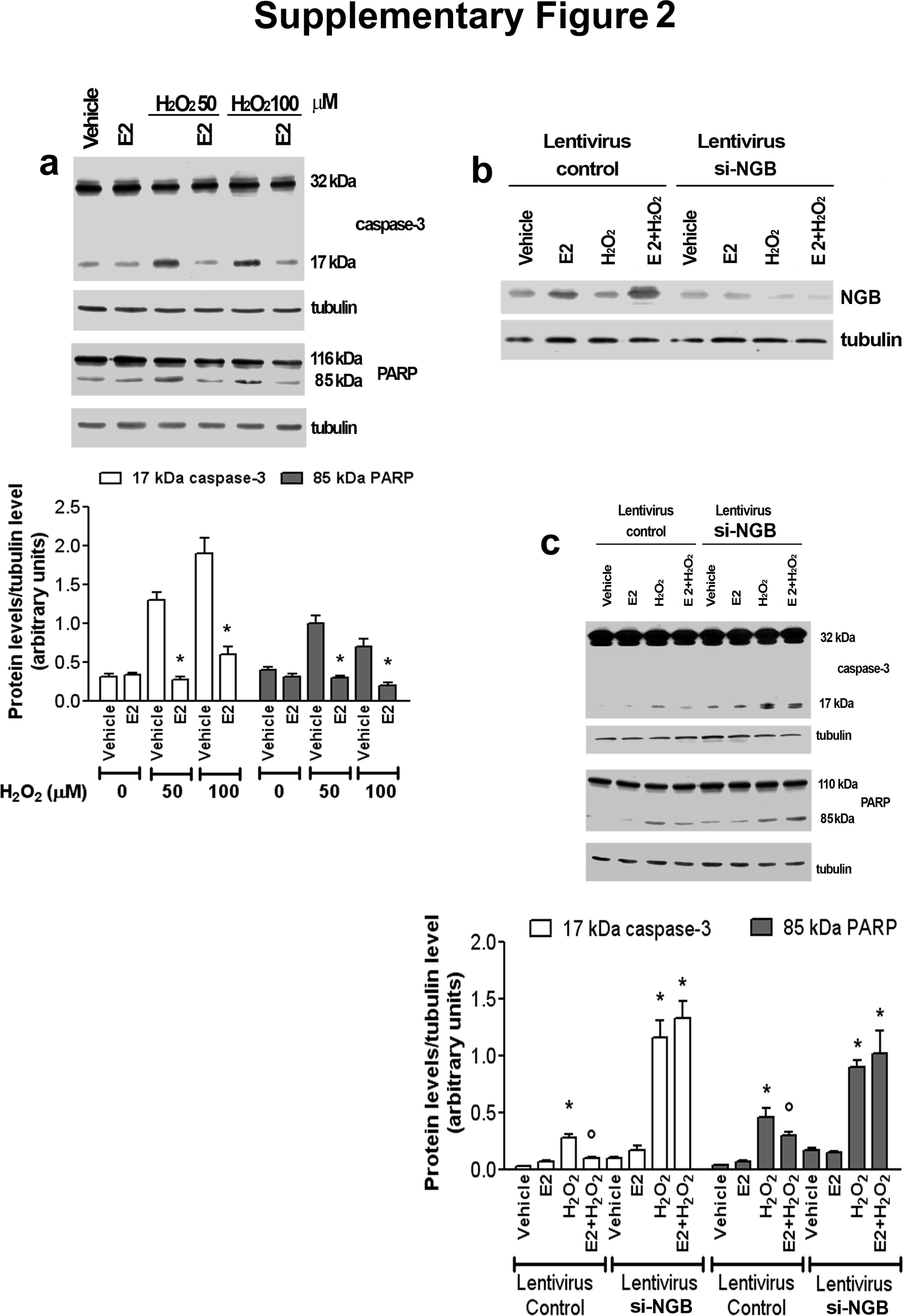

Supplement: Supplementary Figure 2 [file cddis2014418x2.tif]
