# Supplementary material for: Neuroglobin, a pro-survival player in estrogen receptor α-positive cancer cells
Source: Cell Death Dis. 2014 Oct 9;5(10):e1449–. doi: 10.1038/cddis.2014.418 (PMC4237245; doi:10.1038/cddis.2014.418)
Supplement: Supplementary Figure Legends [file cddis2014418x3.doc]

**Supplementary Figure Captions**

**Supplementary Figure 1 Signal transduction pathway activated by E2 in HepG2 and MCF-7 cells.** Time course analysis of phosphorylated (P) and unphosphorylated AKT, p38, and ERK1/2 in a) HepG2 and b) MCF-7 cells stimulated with E2. The amount of proteins was normalized to tubulin or vinculin levels. Top panels are typical Western blots of three independent experiments. Bottom panels represent the results of the densitometric analyses. Data are means ± SD of three different experiments. Significant differences (*p*<0.001) were determined with ANOVA followed by Tukey-Kramer post-test vs. vehicle (*).

**Supplementary figure 2 Effects of E2 on apoptotic-related proteins in HepG2.** (a) Western blot analysis of caspase-3 activation and PARP-1 cleavage were performed on HepG2 cells stimulated with either the vehicle or pretreated with E2 (10 nM; 24 h) followed by 24h treatment with H_2_O_2_ 50 and 100 μM. b) Analysis of NGB protein levels in HepG2 cells infected with either control or with NGB shRNA lentiviral particles and stimulated with either the vehicle or pretreated with E2 (10 nM; 24 h) then treated with H_2_O_2_ 100 μM (24 h). c) Western blot analysis of caspase-3 activation and PARP-1 cleavage were performed on HepG2 cells infected with either control or with NGB shRNA lentiviral particles incubated with either vehicle or 100 μM H_2_O_2_ in the presence or absence of 24 h E2 (10 nM) pre-treatment. The amount of proteins was normalized to tubulin levels. Top panels are typical Western blots of three independent experiments. Bottom panels represent the results of the densitometric analyses. Data are means ± SD of three different experiments. *p*<0.001 was determined with ANOVA followed by Tukey-Kramer post-test vs. vehicle (H_2_O_2_ 0 μM) (*) and vs. vehicle (H_2_O_2_ 100 μM) (°).
